# Enhancement of mixture pollutant biodegradation efficiency using a bacterial consortium under static magnetic field

**DOI:** 10.1371/journal.pone.0208431

**Published:** 2019-01-04

**Authors:** Ahlem Mansouri, Chiraz Abbes, Ramla Ben Mouhoub, Sihem Ben Hassine, Ahmed Landoulsi

**Affiliations:** 1 University of Carthage, Biochemistry and Molecular Biology Lab of Faculty of Sciences, Risks Related to Environmental Stress, Struggle and Prevention (UR17ES20), Bizerte, Zarzouna, Tunisia; 2 Laboratory of Environmental Analytical Chemistry, University of Carthage, Faculty of Sciences of Bizerte, Zarzouna, Tunisia; National Institute of Technology Rourkela, INDIA

## Abstract

One of the main challenges of bioremediation is to define efficient protocols with low environmental impact and high removal rates, such as static magnetic field (SMF). The aim of this study was to evaluate the effect of SMF exposure on the biodegradation rate of a mixture of pollutants using three bacterial strains which were isolated and identified from the Bizerte lagoon: *Pseudomonas stutzeri* LBR (KC157911), *Cupriavidus metallidurans* LBJ (KU659610) and *Rhodococcus equi* LBB (KU743870). To recognize the improvement role of SMF, the culture was submitted to a pre-treatment with SMF with an induction equal to 200 mT for 5 hours, after that the degradation experiment was followed with individual strains and with a consortium. Results showed an increase by 20% in the growth of the exposed bacterial population compared to controls, and 98% of biodegradation of DDT and 90% for BaP after 30 days of follow-up. This encouraging data opens new perspectives for a bioremediation bioprocess using SMF.

## Introduction

Sites with mixed pollution of more than one category of contaminant may be challenging candidates for bioremediation [1]. However, they allow for growth of bacterial strains adapted to the types of contaminants they are exposed to. Therefore, investigations of bacteria isolated from such highly polluted sites might provide information on microbial adaptability in these conditions. These strains are able to interact with multiple contaminants and use the different pollutants as sources of carbon and energy. For that reason they are of particular interest for bioremediation processes [2]. In this context, Bizerte lagoon (Northern Tunisia) has been subjected to large quantities of pollutants over many decades; the majority of these chemicals are persistent, toxic and recalcitrant. Several studies have reported the effect of direct and indirect discharges of urban and industrial wastes, which led to a chemical contamination of the lagoon by various toxic compounds, such as organochlorine pesticides, polycyclic aromatic hydrocarbons (PAHs), heavy metals and organotins [3–4].

Among these pollutants, the major compounds that are widely distributed in Bizerte lagoon are PAHs and pesticides. Included in their toxic effects, are mutagenic, teratogenic or carcinogenic properties. The US Environmental Protection Agency has proposed some of them as a priority pollutant [5]. Two recalcitrant xenobiotics that has caused several toxicological and ecotoxicological problems to the environment and human health are the 1,1,1-trichloro-2,2-bis(p-chlorophényl)ethane (DDT) and the benzo(a)pyrene (BaP) and their residues [6].

The bioremediation of multi-polluted areas is a complicated process [1–7–8] because of the presence of different pollutants that may produce contradictory effects on the degradation process [9–10–11–12]. This is why we are interested in the selection of the bacteria that are able to degrade a mixture of pollutants and are the most adapted to this environment.

Many organisms have the capacity to degrade pollutants in simple and mixed forms; furthermore, the biodegradation of DDT was possible by various bacteria such as *Alcaligenes eutrophus* A5, *Bacillus* sp. BHD-4, *Pseudomonas putida*, *Pseudomonas* sp. and *Serratia marcescens* DT-1P [6]. Studies carried out on BaP-degrading bacteria have shown that it was possible for bacteria to degrade BaP when grown with BaP as a unique carbon source or under an alternative carbon source in liquid culture experiments. Some strains where this has been documented include *Sphingomonas paucimobilis* EPA 505 [13], *Agrobacterium* sp., *Pseudomonas* sp. and *Burkholderia* sp. [14]. BaP has been reported to be degradable by other bacterial strains, including *Rhodococcus* sp., and a mixed culture of *Pseudomonas* and *Flavobacterium* species [15].

Later, several studies were conducted in order to improve the biodegradation efficiency of isolated strains. One of the novel methodologies developed was the combination of the static magnetic field (SMF) effect and other technology. Recently, it was shown that SMFs can improve the energy density of magnetite / polypyrrole nanocomposite capacitors by 10 fold [16]. Thus a magnetic field of 9 T has generated a high magnetoresistance in the nanocomposites of cobalt ferrite (CoFe_2_ O_4_) / polyaniline (PANI) [17].

In biodegradation; electromagnetism may interact with organisms in both a negative and positive manner that includes acceleration of growth and metabolism [18]. The effect of SMF in biodegradation of organic pollutants has improved waste water treatment [19]. It was demonstrated that such a combination enhances the removal of organic compounds by a factor of 70% compared to control treatment. Thus, the combination between SMF treatment and the use of different bacterial strains contributes well to the bioremediation measure, and has proven to be a promising means for clean-up of highly polluted sites [20].

The present study summarizes the isolation and identification of bacterial strains capable of using the mixture of DDT and BaP as the ultimate source of carbon and energy. The main objectives of this research were: i) to select bacteria capable of degrading the mixture of BaP and DDT from the water of the Bizerte lagoon; ii) to reveal the potential biodegradation of a DDT and BaP mixture over a 30 day period; and iii) to improve the microbial biodegradation kinetics and yield by partnering it with SMF. The results will provide a new insight into the remediation of mixed polluted sites.

## Materials and methods

### Study area

Water samples were taken without any required permit from a highly polluted area: the Bizerte lagoon, a public land located in Northern Tunisia (latitude: *37* °*8'*–*37*°*14'* N, longitude: *9°46'–9°56'* E).

Bizerte Lagoon extends over a surface area of approximately 150 km^2^, with a maximal width of 11 km and maximal length of 13 km, and is connected with the sea by a channel of 6 km in length. The lagoon is also connected in its part West with the lake Ichkeul [21]. It is an open dumping disposal site, which has been affected by many anthropogenic pressures including urbanization, industrial and agricultural activities.

The study was conducted in the laboratory and there are not any ethical or legal restrictions on sharing of all the data.

### Media

Luria–Bertani (LB; Difco, USA) medium contains (per L): 10 g NaCl, 10 g peptone, 5 g yeast extract, with or without 20 g agar in distilled water. The solution pH was adjusted to 7. M9 medium contains (per L) 200 ml M9 salt (12.8 g Na_2_HPO_4_.7H_2_O, 3 g KH_2_PO_4_, 0.5 g NaCl, 1g NH_4_Cl, 200 mL distilled water), 2 mL MgSO4 1M, 0.1 mL CaCl_2_ 1 M and the solution pH was adjusted to 7.

### Isolation of DDT and BaP degrading bacteria

The enrichment and isolation of DDT and/or BaP degrading bacteria were carried out using the mixture of BaP and DDT as the only source of carbon and energy. Two mL of lagoon water were used to inoculate 18 mL of M9 minimal medium containing a final concentration of 50 mg/L DDT and/or 50 mg/L of BaP. A culture was incubated with shaking at 200 rpm at 30 °C for 30 days in the dark. Every 6 days, 2 mL of the culture were taken and added in fresh medium. A non-inoculated flask without carbon source served as control. The aliquot (1 mL) of the sample dilution was spread on a culture medium at 30 °C for 48 hours. Then, pure strains were isolated. Afterword, pure bacterial strains were grown in M9 medium supplemented with DDT and BaP to preserve their degradation ability.

### Phylogenetic analysis of *16S*r DNA sequence

Genomic DNA was extracted with FERMENTAS Gene JET Genomic DNA purification kit. *16S* rDNA was amplified in PCR using the genomic DNA as template. The bacterial universal primers were as follows: 8 F (5’ AGAGTTTGATCCTGGCTCAG 3’), 1489 R (5’ TACCTTGTTACGACTTCA 3’). The amplification was conducted as described by Mansouri and co-workers [22]. Sequences were compared to those present in the databank using the BLAST program (www.ncbi.nlm.nih.gov/BLAST/) and aligned with the DAMBE program (version 5.5.24). A phylogenetic tree was obtained using the Mega software (version 6.06) and the neighbour-joining method. The confidence of the phylogenetic tree was analysed by the bootstrap method of 1000 trials.

### Static magnetic field set-up

The bioreactor used in the experiment was a dark double phial, horseshoe-shaped, with a pair of neodymium magnets on the ends (dimension of neodymium ring magnets: 20 mm outer diameter, 5 mm inner diameter and 10 mm thick). The bioreactor was 45 mm in diameter and 200 mm in height, with a wall thickness of 0.5 mm. The total reaction volume was 50 mL.

To create SMF, samples in the bioreactor were placed in the gap where flux density values remain approximately the same, meaning that samples were exposed to the same intensity over their volume. A GM08 Gauss meter was used to measure magnetic flux density. The samples were maintained, continuously shaken for homogeneity. Temperature of 30 °C was maintained inside the bioreactor by water circulation, using an incubator system composed of a peristaltic pump and resistance. The control setup was identical but with the absence of magnets. The experiments were done over 5 days with individual strains. The follow-up was done during 30 days for the consortium, as explained above, by combining with a pre-treatment using a magnetic field exposure for 5 hours. Both SMF and control samples were done in triplicates.

### Analysis of DDT and BaP degradation

In order to determine the DDT and the BaP mixture degradation ability, strains LBR, LBB and LBJ were inoculated in 50 mL of a mineral medium with the initial DDT and BaP concentration of 10 mg/L. The different compositions used in the degradation of PAH were (i) M9 medium with DDT and BaP, in the presence of bacteria strain (individual strain or with consortium) (ii) M9 medium + DDT + BaP and (iii) M9 medium + bacteria strain where (ii) and (iii) served as controls.

DDT and BaP were extracted three times using dichloromethane (DCM) after acidification to pH 2.5 with 1 N HCl. The extracts were filtered through a gradient of florisil and anhydrous sodium sulphate and condensed to 1 mL for the chromatography analysis of DDT and BaP concentration. The condensed sample was filtered through 0.2 mm syringe filter and was quantitatively analysed using high performance liquid chromatography (HPLC, with C 18 column (Zorbax Eclipse, C18 5μm, 4.6*250 mm), Agilent Technologies (USA) with UV–VIS detector connected to the WINCHROME software, under wavelength 238 nm for DDT and 294 nm for BaP quantification [23, 24], and acetonitrile to deionized water ratio (7:3, V/V) at flow rate of 1 mL/min. Quantification was based on the internal standard, DDT and BaP calibration curves.

### Statistical analysis

All statistical analyses were performed using STATISTICA 7.1. Data was initially checked for homogeneity of variances. Then, an ANOVA, followed by the post hoc Tukey test were performed to assess differences between treatments and a significance level of 0.05 was used. All experiments were repeated independently 3 times (three biological replicates) with independent inoculation of bacteria.

## Results

### Characteristics and molecular identification of isolates

Following the isolation technique, three morphologically distinct bacterial strains were isolated from the Bizerte-lagoon. The isolates named LBR, LBB and LBJ were identified.

**Fig 1:**
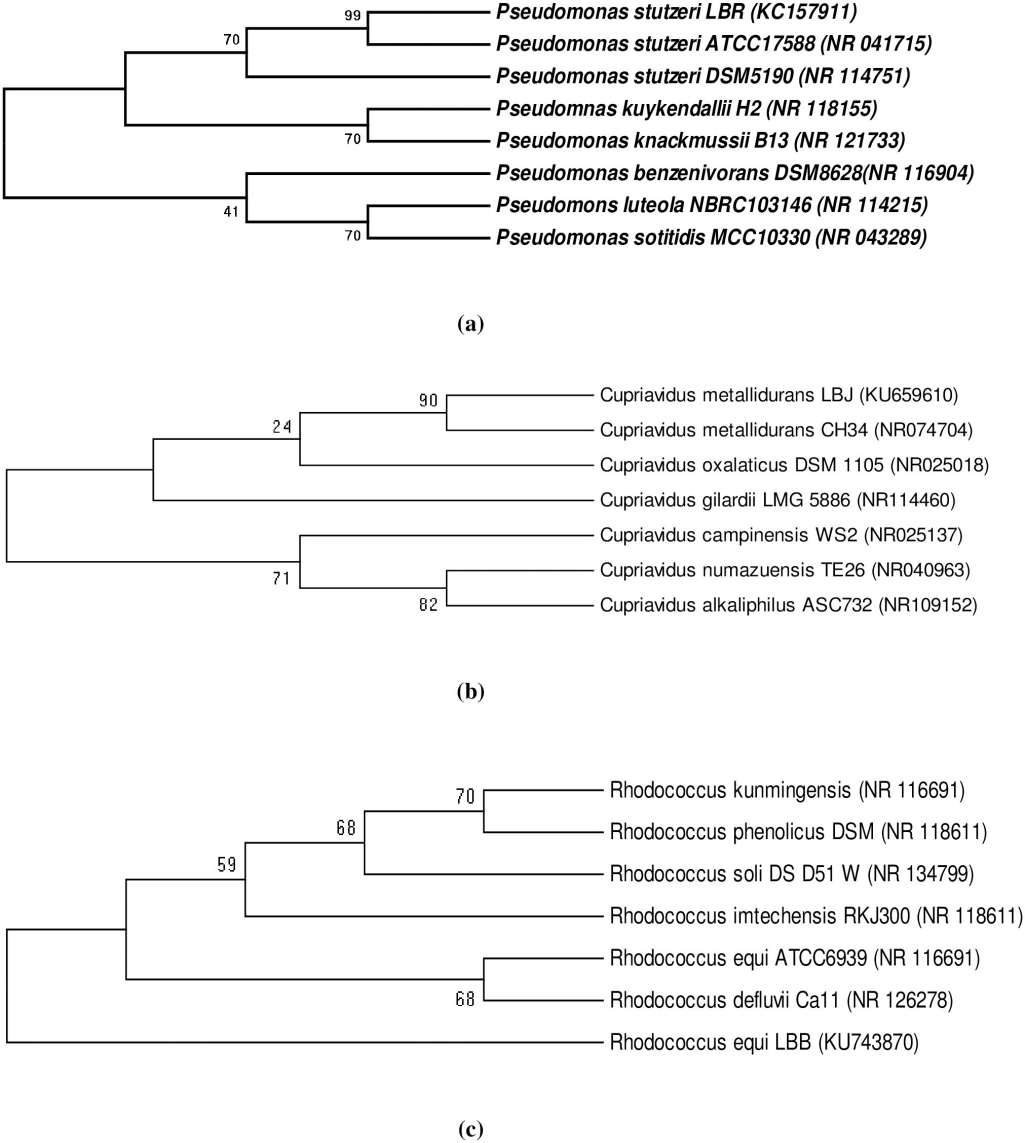
Neighbor-joining phylogenetic tree based on *16S*r DNA of the isolates *Pseudomonas stutzeri* LBR (a), *Cupriavidus metallidurans* LBJ (b) and *Rhodococcus equi* LBB (c). The tree was generated with 1000 replicates and the numbers at the nodes represent values for interior-branch test.

The *16S* rDNA sequences show that the LBR, LBJ and LBB isolates are closely related to the strains *Pseudomonas stutzeri* ATCC 17588 (NR-041715.1), *Cupriavidus metallidurans* CH34 (NR-074704.1) and *Rhodococcus equi* ATCC6939 (NR-116691.1), respectively, with 100, 99 and 93% of identity. Therefore, the isolates were named *Pseudomonas stutzeri* LBR (P. *stutzeri* LBR) (Fig 1a), *Cupriavidus metallidurans* LBJ (C. *metallidurans* LBJ) (Fig 1b) and *Rhodococcus equi* LBB (R. *equi* LBB) (Fig 1c). The 16Sr DNA sequences of strains were deposited in the GenBank database, respectively, under the accessions numbers: KC157911, KU659610 and KU743870.

Because microbial degradation is the major route to remove pollution from contaminated areas, screening degrading strains with the ability to remove specific pollutant mixtures is an important novel means of the bioremediation process; the isolation of effective bacteria is a crucial step for the removal of mixed pollutants [25– 26]. The degradation capacity of all isolated strains has been demonstrated in the literature.

*P*. *stutzeri* is a denitrifying bacterium, and considered as a model organism for denitrification research [27]. A recent study showed that *P*. *stutzeri* has the capacity to degrade phenanthrene, pyrene and benzo(a)anthracene in a bioreactor with 95,78 and 82% of removal, respectively [28]. The studies mentioned above show that *P*. *stutzeri* was able to degrade low and high molecular weight PAHs and pesticides belonging to the organophosphate family. In this work, it has been shown, for the first time, that *P*. *stutzeri* LBR was able to degrade BaP and DDT.

The strain LBJ, identified as *Cupriavidus metallidurans*, was reported to be resistant to several heavy metals. It provides genes encoding resistance functions to heavy metals ensured by mega-plasmids and it was demonstrated that this strain was able to remove Cd and Pb from contaminated soil [29]. Jones and colleagues [30] used substrate enrichment, and pyrosequencing to identify the genera *Cupriavidus* as the strains most related to benzo(*a*)pyrene co-metabolism in PAH-polluted soil.

The isolate LBB, related to *Rhodococcus equi* was demonstrated to be able to degrade hexane and other aromatic compounds [31]. In 2011, Song and colleagues showed that the strain *Rhodococcus* sp.P14 was the first bacterium identified, of the genus *Rhodococcus*, which has the ability to degrade high molecular weight PAHs [25]. Recently, its ability to degrade DDT and its metabolites was also demonstrated [32]. The present study was the first demonstration that *Rhodococcus* can degrade DDT and BaP in a mixture.

### Biodegradation efficiency of DDT and BaP by individual strains without SMF

**Fig 2:**
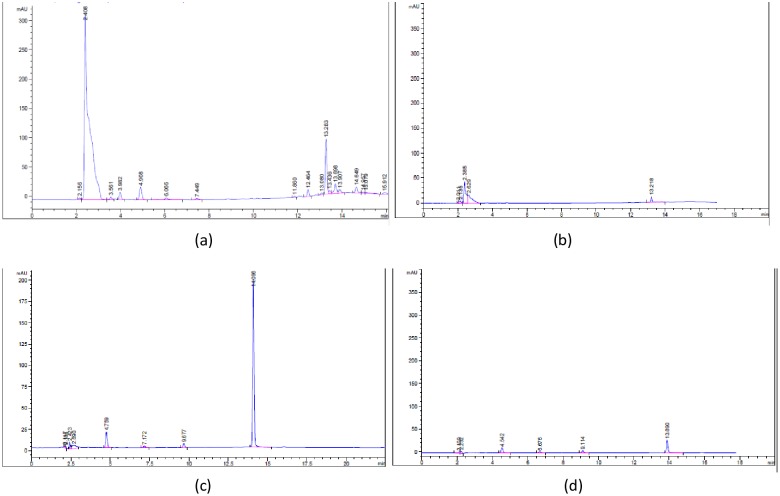
Comparison between the chromatograms of DDT at the initial time (a) and at 30 days of monitoring (b) and BaP before treatment (c) and at the end of the treatment (d) resulting from the kinetics of biodegradation of *Pseudomonas stutzeri* LBR during 30 days.

The bacteria strains of *P*. *stutzeri* LBR, *C*. *metallidurans* LBJ and *R*. *equi* LBB were treated separately with 10 mg/L DDT and 10 mg/L BaP for 30 days in mineral salt media at pH 7.0 and at 30 °C, to screen for degradation ability of each strain using a high performance liquid chromatography with UV detection (Fig 2). As shown in Table 1, the isolated bacteria *P*. *stutzeri* LBR degraded almost 87% of the added DDT in 30 days, and 83% of the added BaP.

**Table 1:** Biodegradation efficiency of DDT and BaP by individual strains without SMF for 30 days.

	*P*. *stutzeri* LBR	*C*. *metallidurans* LBJ	*R*. *equi* LBB
	Percentage of biodegradation (%)		
**DDT**	87 ± 1.52	79 ± 2,44	68 ± 2.71
**BaP**	83 ± 0.95	57 ± 1.79	50± 2.49

For *C*. *metallidurans* LBJ, the biodegradation of DDT and BaP was 79% and 57% of initial quantity of DDT and BaP, respectively. Meanwhile, *R*. *equi* LBB was able to degrade around 68% of DDT and only 50% of BaP after 30 days of incubation (Table 1). In this research we used two types of control: M9 medium with DDT + BaP and M9 medium with bacteria strain, in both cases no significant decrease in the initial concentrations of DDT and BaP and no bacterial growth was recorded.

### Biodegradation efficiency of DDT and BaP by individual strains with SMF

To investigate whether SMF had an effect on the degradation ability of the DDT-BaP mixture by *P*. *stutzeri* LBR, *C*. *metallidurans* LBJ and *R*. *equi* LBB, the experiment was carried out with individual strains in mineral medium with DDT and BaP as sole carbon sources at 10 mg/ L at 30 °C for 5 days, with a pre-treatment of 5 hours of SMF at 200 mT (Table 2).

**Table 2:** Biodegradation efficiency of DDT and BaP by individual strains with and without SMF for 5 days.

	*P*. *stutzeri* LBR			*C*. *metallidurans* LBJ			*R*. *equi* LBB		
	Percentage of biodegradation (%)		Gain factor	Percentage of biodegradation (%)		Gain factor	Percentage of biodegradation (%)		Gain factor
	Without SMF	With SMF		Without SMF	With SMF		Without SMF	With SMF	
**DDT**	16 ± 0.45	34.5 ± 1.32	2.15	12 ± 1.46	29.8 ± 2.32	2.48	10 ± 1.48	30 ± 2.31	3
**BaP**	9 ± 0.15	21.5 ± 2.13	2.38	6.8 ± 0.98	18.2 ± 1.02	2.67	9.3± 0.45	19 ± 3.23	2

With individual strains and SMF, the results showed that the degradation rate was increased for all tested bacterial species, therefore, after 5 days of follow-up, 200 mT SMF increased the degradation capacity of DDT and BaP above two fold, by *P*. *stutzeri* LBR, compared to control treatment (without SMF). Indeed, the disappearance of 34.5% and 21.5% of DDT and BaP, respectively, was recorded in the presence of SMF after only 5 days, against 16% and 9% in the control samples (Table 1). In presence of the strain *C*. *metallidurans* LBJ and 200 mT of SMF, the biodegradation rate was 29.8% and 18.2% for DDT and BaP, respectively, compared to 12% and 6.8% in the control samples (Table 1). For *R*. *equi* LBB, under SMF intensity of 200 mT, a biodegradation about 30% of DDT and 19% of BaP, was obtained compared to 10% and 9.3%, respectively, in the control samples (Table 2).

This is a very interesting result because it will allow for the total biodegradation of each individual strain, in less time and at lower cost than non-exposed samples.

### Biodegradation efficiency of DDT and BaP by consortium with SMF

**Fig 3:**
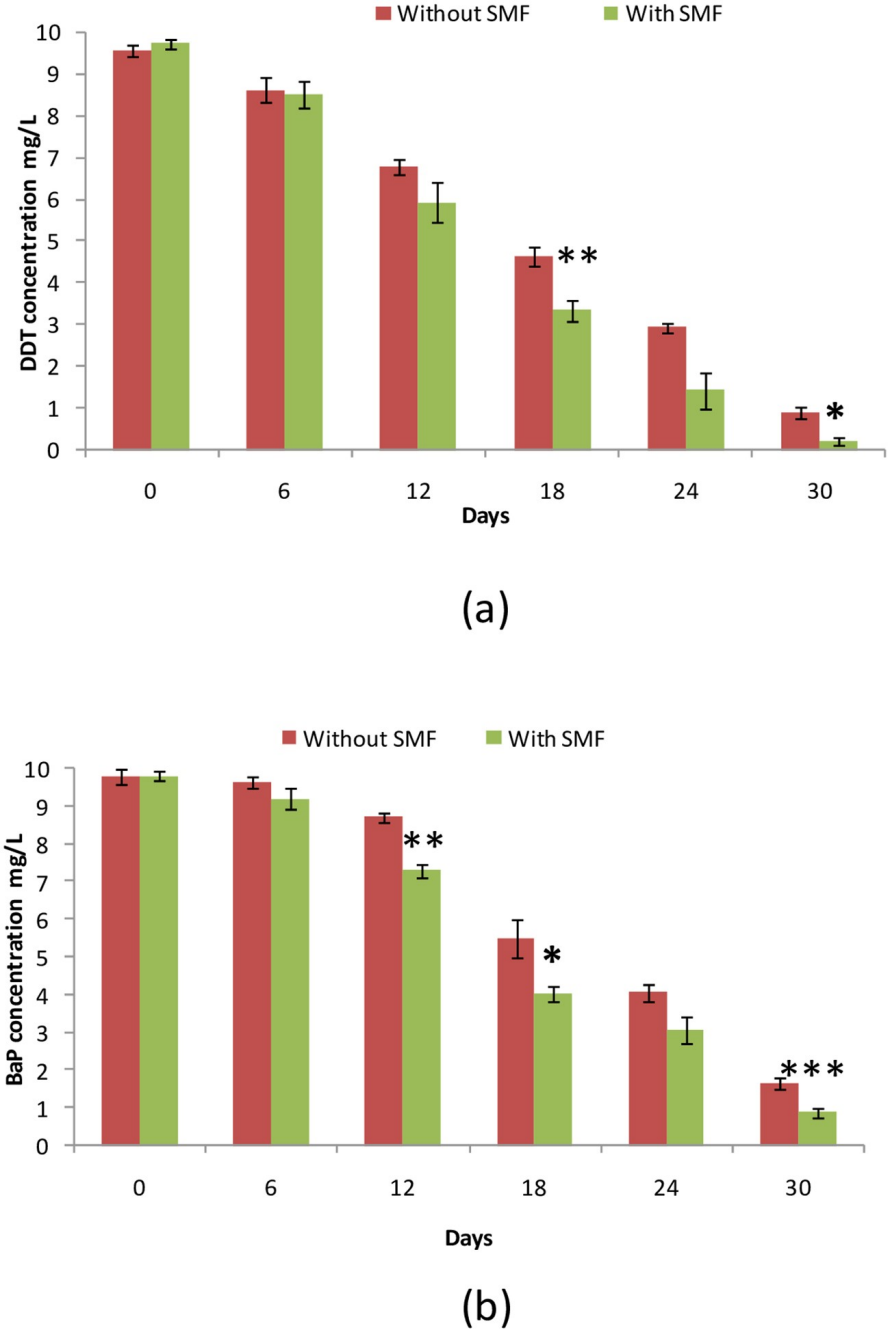
Biodegradation kinetics of DDT (10 mg / L) (a) and BaP (10 mg / L) (b) in a mixture with a consortium consisting of *Pseudomonas stutzeri*, *Cupriavidus metallidurans* and *Rhodococcus equi* for 30 days, with and without SMF. Asterisks indicate the signification *: significant difference (p<0.05), **: very significant difference (p<0.01), ***: highly significant difference (p<0.001).

When biodegradation was done with the bacterial consortium composed with *P*. *stutzeri* LBR, *C*. *metallidurans* LBJ and *R*. *equi* LBB for 30 days, and to avoid the competition between the different bacterial strains, biodegradation experiments were made during 30 days of monitoring with a pre-treatment with SMF of 5 hours. Results indicated that we had reached 98% biodegradation of DDT and 90% for BaP with SMF compared to 90% and 81%, respectively, without magnetic field (Fig 3).

The effect of static magnetic field on the pollutants was studied and we found that the biomolecules are not affected by the static magnetic field. This has been illustrated by several studies [18–22].

The biomass content of both control and exposed samples was monitored for 30 days (data not showed). We observed an increase of the biomass for all exposed strains, which was significantly higher than in the control samples. The increase of biomass content was proportional to the decrease of the DDT and BaP concentrations.

Under our experiment conditions, the SMF 200 mT appeared to allow stimulation of organic pollutant degradation and to promote growth of the bacterial population by 20% compared with the control.

## Discussion

Several efforts have been made to improve the biodegradation effectiveness whose SMF effect. Generally, the impact of SMF depends on its intensity, for instance at more than 1 T, the physiological processes in organisms will be inhibited [33]. However a weak SMF may also stimulate the biodegradation of wastewater [19]. In a previous study, Filipic and colleagues proved that exposure to low intensities of 17 mT SMF could affect the activity of physiological processes in two ways: it could stimulate the activity of certain enzymes for biodegradation of organic compounds and repress other growth enzymes in *Escherichia coli* and *Pseudomonas putida* [18]. According to Křiklavová and co-workers, the membrane permeability that was affected by the electromagnetic field might result in alterations of ion transport of substrates [34]. As a consequence, cellular metabolism was stimulated to increase DDT and BaP uptake and degradation. These modifications in cell membrane altered its ionic composition and also the biochemical and mechanical transport properties which could enhance specific enzyme activity [35].

The role of SMF in the biodegradation of organic pollutants has been studied with several bacterial strains [22– 36]. Positive effects were obtained with an improvement of wastewater treatment [36]. Moreover, the influence of moderate SMF on bacterial physiology is still poorly understood, and those mechanisms responsible for the effects of SMF on wastewater treatment processes are still unknown. Potenza and colleagues reported that the static high magnetic field (300 mT) may influence cell growth and gene expression. These biological effects may be associated with DNA stability of disturbance by direct interaction with DNA or by potentiating the activity of oxidizing radicals [37]. In another study, they observed a remodelling of membrane lipid composition of *Salmonella typhimurium* to maintain an optimum level of fluidity under SMF (200 mT) [38].

In biodegradation, Křiklavová et al. observed that SMF increased phenol degradation by a factor of 35%, in the case of *Rhodococcus erythropolis* and accelerated the growth about 28% in a fed-batch reactor [34]. According to Zaidi et al., SMF had two enhancements which lead to an increase in the efficiency of water and wastewater treatment performances [39]: First, it has the potential to improve physical performance, in terms of solid-liquid separation. Secondly, it changes the biological properties through the improvement of bacteria activity.

## Conclusion

Our investigation focused on the application of SMF in environmental engineering, in order to improve the bioremediation of a mixture of pollutants. The Bizerte lagoon, a high polluted site, was the source of three bacterial strains able to degrade a mixture of DDT and BaP: *Pseudomonas stutzeri* LBR, *Cupriavidus metallidurans* LBJ and *Rhodococcus equi* LBB. The bacteria strains of *P*. *stutzeri* LBR, *C*. *metallidurans* LBJ and *R*. *equi* LBB were able to remove 87, 79 and 68% of added DDT, and 83, 57 and 50% of added BaP in 30 days, respectively. With individual strains and under an induction equal to 200 mT of SMF, our results showed that after 5 days of follow-up, the degradation rate was increased for each of the tested bacterial species. This will allow us to achieve total biodegradation with each individual strain, in less time and at lower cost, compared to the control samples. Furthermore, when biodegradation was carried out with the bacterial consortium over 30 days, results showed that 98% of biodegradation of DDT and 90% of BaP under 200 mT of SMF.

This is the first published research that demonstrated that the combination of isolated biodegrading bacteria and the metabolic effect of 200 mT SMF is a promising new method for DDT and BaP removal from polluted environments. However, further studies with the combination of biodegradation ability of those isolates and SMF in polluted water and sediment needs to be conducted carefully in the future.

## Supporting information

S1 Fig Experimental setup for the fed-batch reactor used for DDT and BaP biodegradation. The control setup (without a static magnetic field) was identical but with the absence of magnets. 1:Bioreactor, 2: Magnets, 3: Probes for monitoring parametrs (cells count, monitoring O_2_, temperature and pollutants analyses), 4: Aerator, 5: incubator system, 6: Water circulation system with peristaltic pumps.
(DOCX)
