# Supplementary material for: Enhancement of mixture pollutant biodegradation efficiency using a bacterial consortium under static magnetic field
Source: PLoS One. 2019 Jan 4;14(1):e0208431. doi: 10.1371/journal.pone.0208431 (PMC6319723; doi:10.1371/journal.pone.0208431)
Supplement: S1 Fig — The control setup (without a static magnetic field) was identical but with the absence of magnets. 1:Bioreactor, 2: Magnets, 3: Probes for monitoring parametrs (cells count, monitoring O2, temperature and pollutants analyses), 4: Aerator, 5: incubator system, 6: Water circulation system with peristaltic pumps. (DOCX) [file pone.0208431.s001.docx]

**Figure:** Experimental setup for the fed-batch reactor used for DDT and BaP biodegradation. The control setup (without a static magnetic field) was identical but with the absence of magnets.

1:Bioreactor, 2: Magnets, 3: Probes for monitoring parametrs (cells count, monitoring O_2_, temperature and pollutants analyses), 4: Aerator, 5: incubator system, 6: Water circulation system with peristaltic pumps

**Assessment of pollutants toxicity for the bacteria strains**

The microbial respiration (consumption of oxygen or release of CO_2_) is used for chemical fate assessment and especially for the revelation of precise biodegradation rates.

This procedure, involve the reaction of the resazurin which is a biomarker of biological activity, in order to use the reducing power of NADH and NADPH produced during chemical catabolism to produce resorufin (reduced form of resazurin) which have a fluorescent ability when excited by green light wavelength, thus the increasing of this fluorescence is correlated to the organic matter consumption.

Micro plates (96 plates) were used to quantify cellular activity in the presence of a large game of concentration of the mixture of DDT, BaP and metals. Metal mixture was prepared under the protocol proposed by Mowat and bundy. Metal concentrations tested were the same in Bizerte lagoon: Zn 11 mg/L, Cu 3.70 mg/L, Pb 2.80 mg/L, Cd 0.20 mg/L.

Before testing, bacteria strain cells were incubated overnight in Luria Bertani medium at 30 °C under agitation at 250 rpm. To remove residual carbon, cells were then washed with three successive centrifugations in saline solution (MgSO_4_ at 10^−2^M, pH 7.4) at 6400 g for 10 min. Finally, the cells were re-suspended in sterile M9 medium to obtain the freshly washed inoculums.

**Result:**

The toxicity evaluation of DDT, BaP mixture and a metals cocktail on *Pseudomonas stutzeri* LBR, *Cupriavidus metallidurans* LBJ, and *Rhodococcus equi* LBB was done by a monitoring of oxygen consumption. The inhibition rate of respiration has been expressed as IC_50_ value for each strain in the presence of DDT, BaP mixture and metals. The IC_50_ was 42.5 mg/L (±6.1 mg/L), 40.2 mg/L (± 3.8 mg/L) and 37.6 mg/L (± 4 mg/L) respectively for *Pseudomonas stutzeri* LBR, *Cupriavidus metallidurans* LBJ, and *Rhodococcus equi* LBB.

Given those results, and to ensure maximum activity of all strains, we chose to spike mineral medium with final concentration of 10 mg/L for both DDT and BaP.
